# Ethical content in artificial intelligence systems: A demand explained in three critical points

**DOI:** 10.3389/fpsyg.2023.1074787

**Published:** 2023-03-30

**Authors:** Ana Luize Corrêa Bertoncini, Mauricio C. Serafim

**Affiliations:** AdmEthics – Research Group in Ethics, Virtues, and Moral Dilemmas in Administration, Administration Graduate Program of the Administrative and Socioeconomic Sciences College, Santa Catarina State University, Florianópolis, Brazil

**Keywords:** artificial intelligence, ethics, autonomy, right of explanation, value alignment, moral agency

## Abstract

Artificial intelligence (AI) advancements are changing people’s lives in ways never imagined before. We argue that ethics used to be put in perspective by seeing technology as an instrument during the first machine age. However, the second machine age is already a reality, and the changes brought by AI are reshaping how people interact and flourish. That said, ethics must also be analyzed as a requirement in the content. To expose this argument, we bring three critical points - autonomy, right of explanation, and value alignment - to guide the debate of why ethics must be part of the systems, not just in the principles to guide the users. In the end, our discussion leads to a reflection on the redefinition of AI’s moral agency. Our distinguishing argument is that ethical questioning must be solved only after giving AI moral agency, even if not at the same human level. For future research, we suggest appreciating new ways of seeing ethics and finding a place for machines, using the inputs of the models we have been using for centuries but adapting to the new reality of the coexistence of artificial intelligence and humans.

## 1. Introduction

Artificial intelligence (AI) is changing people’s lives in ways never imagined before. Machine learning, robots, algorithms, and autonomous vehicles, among others, carry out productive activities and give sophisticated solutions to improve society (Awad et al., 2018; Hooker and Kim, 2019a). The main goal is to make life easier and more pleasant (Kim et al., 2021), promote well-being, and cause no harm or, at least, minimize it (Awad et al., 2018). However, this equation is not so simple, and troubles emerge from the definition of what artificial intelligence means and, in practical terms, how AI works (Tegmark, 2017). It reflects the complexity of delimiting AI’s boundaries since the new technologies’ benefits, opportunities, and threats share the scene in the still unknown consequences.

As an opportunity, we can mention some potential outcomes like reducing social evils; but machines may also substitute humans in dangerous or unpleasant activities (Anderson and Anderson, 2011). As a critical property of Industry 4.0, it enabled using a significant amount of raw data in knowledge, reducing cost, increasing quality, and improving work conditions (Kim et al., 2021). The first machine age introduced the innovations responsible for substituting human muscle power, creating actual modern life, and sharply bending up the curve of human history to several people and developments never seen before. Now, the world is entering the second machine age, where technology is creating a mental power that is expected to overcome past limitations and lead us to new levels of improvement (Brynjolfsson and McAfee, 2016). So, it makes perfect sense to imagine AI that imitates a broader notion of intelligence that contains wisdom rather than an instrumental view of intelligence (Kim and Mejia, 2019).

Nonetheless, there is still no consensus on what artificial intelligence is and whether the technical challenges will be overcome to achieve a strong type of AI. Nevertheless, these technologies have been adopted in a wide range of domains (Awad et al., 2018; Hooker and Kim, 2019a; Anagnostou et al., 2022; Ashok et al., 2022; Miller, 2022; Munn, 2022), while our understanding of its ethical and societal implications is trivial. In addition, one may see how the evidence proves that unintended consequences may happen more often than expected, despite good intentions (Coeckelberg, 2020). Besides, not just the range and implications of the technical aspects are under evaluation. One can easily understand artificial intelligence as part of computer science and matter for engineering studies; still, the attempt to make AI decisions human-like also prompts cognitive scientists. AI also had become a prolific area of research about the human mind and rekindled century-old discussions regarding decision-making, human actions, rationality, and cognition, among others (Franklin, 2014). In this regard, the long-lasting debate regarding human intelligence was expanded by artificial agents trying to replicate it (Hooker and Kim, 2019b); and one of the most fruitful is ethical questioning.

In this context, if we were concerned about developing ethics for humans using machines, now we urgently need to discuss ethics for machines. By seeking to imitate human behavior, AI contrasts with traditional technologies, and, in this regard, the perspective of analyzing ethics changed. From the instrumental standpoint, machines are not more than tools used by humans, so the ethics rest in the individuals using the machines and involve their proper and improper use (Anderson and Anderson, 2011). This scenario fits the first machine age, marked by the Industrial Revolution when machines substituted the human’s muscle power (Brynjolfsson and McAfee, 2016). It means that all trade-offs and moral dilemmas drew in were humans’ responsibility, and the liability is easy to trace back. But what happens when the decision-making recalls solely on the machines? To answer this question, the changes caused by AI are reshaping how people interact and flourish while improving our lives (Kim and Mejia, 2019); that said, ethics is one of the features of human life that should be reconsidered.

To expose this argument, first, we introduce why we should focus on AI ethics. Following, we guide the debate with three critical points: autonomy, right of explanation, and value alignment. Our argument shows that these three crucial points must be considered when analyzing AI’s mimetic process to replicate human-like actions and decisions (Anderson and Anderson, 2011) and, in consequence, describe why ethics must be part of the system’s content, not just in the principles to guide users. In the last part of this paper, we show how these points lead the discussion to a reflection on AI’s agency. We propose that ethical questioning must be solved only after the AI’s moral agency is clarified.

## 2. Why do AI ethics matter?

Artificial intelligence reaches most of anyone that uses modern technologies, but the whole social fabric will undoubtedly be influenced somehow by its outcomes (Franklin and Ramsey, 2014). Notwithstanding, these advancements raise a host of societal questions as far as the technology and its algorithms silently define our lives, for example, in job promotions, loan offerings, and products consumers might see (Martin, 2019; Kim et al., 2021). Areas like education can easily be automated, and even medical technologists have been overtaken by machines (Hooker and Kim, 2019a). These are cases in crucial evaluations already under discussion, such as life-and-death medical decisions. The excuse to let these interventions into our lives is that future improvements are expected to reduce inequality, poverty, disasters, war, etc. Thus, the discussion is not about technology; it is about our future (Tegmark, 2017). This context would be enough to see AI’s relation to ethics but going deeper into its background will ensure we are not overlooking the situation.

Many technologies have changed society before. However, for the first time, the technology created might substitute the creators in exclusively human activities and not just collaborate with them, as in the previous evolutions (Brynjolfsson and McAfee, 2016). AI is considered the non-biological type of intelligence that represents the last critical point of life, the technological phase (life 3.0) when both software and hardware can be designed by themselves. This capacity started when the computing data process evolved and added the learning ability, permitting algorithms to learn (Tegmark, 2017). The machine learning ability, born from Arthur Samuel’s checker playing program, turned machines able to evolve from algorithms (Franklin, 2014). Nonetheless, despite this capacity, controversies emerge from the fact that algorithms are still just sequences of instructions that guide machines, or whatever technology it is embedded, into actions using information inputs and giving outputs (Coeckelberg, 2020).

AI’s particular individualities highlight both the computer/technical part and the science side, which helps the scientific area understand human intelligence and replicate it (Franklin, 2014). This cross-disciplinary characteristic reflects the intersection of computability theory from the previous decades and the cognitive revolution. These two critical developments express the moment when the term “artificial intelligence” was coined in 1956 during the seminal Dartmouth Conference (Arkoudas and Bringsjord, 2014). Furthermore, it is crucial to remember that AI is not one technology but a set of them. Classified by their nature, they can be separated into two major buckets (Kim et al., 2021): the strong AI, or General Artificial Intelligence (GAI or AGI), that aims to build human-like technologies with intelligence across domains; and the weak AI, which creates machines that act intelligently without taking a position on whether the AI systems are intelligent in fact (Arkoudas and Bringsjord, 2014; Kim et al., 2021). An alternative classification comprehends AI partners, which assist humans, and AI minds, as the ones aiming to overcome humans (Etzioni and Etzioni, 2017). In this sense, some authors talk about a superintelligent AI intending to improve human beings and even achieve immortality by transferring the human brain to a robot. However, it is still unclear how much the discussions about superintelligence are relevant to the development of this area’s studies (Coeckelberg, 2020).

In addition, the very description of what artificial intelligence means is still controversial. Saying that AI is the technology able to show intelligence through algorithms is too imprecise from a philosophical and ethical viewpoint. The lack of a universal definition is one of the reasons why the intelligence conception used for AI is usually compared to the human one (Coeckelberg, 2020). In this sense, the anthropomorphic illusion^1^ is an explanation for the comparison, represented by the reductionism in the view of human beings, reflected in mechanicism - on the epistemological level - and in utilitarianism - on the ethical level (Bertolaso and Rocchi, 2022). Therefore, if defining intelligence has always been challenging, the modern form of intelligence did not make it easier to follow the idea. Although it is simple to understand that artificial means non-human, artificial intelligence comprehends a broad field dedicated to developing artifacts capable of intelligent behavior in controlled environments and over specific periods (Arkoudas and Bringsjord, 2014). From a different perspective, AI can also be seen as an interdisciplinary approach to understanding, building models, and replicating intelligent and cognitive processes based on computational, mathematical, mechanical, and even biological principles (Kim et al., 2021).

However, one of the problems of AI’s delimitation relies on the inconsistency pervading those underlying concepts: they infer precise and intelligent systems in particular domains, and they do not always mean human intelligence. For example, animals may also show intelligent behavior (Kim et al., 2021). That said, a more comprehensive definition should also consider the demonstration of “intelligence through non-biological/natural processes” (Kim et al., 2021, p. 357). In this connection, Tegmark (2017, p.85) considers intelligence the “ability to accomplish complex goals.” He prefers to use a comprehensive report and inclusive view since there is no undiscussable definition of intelligence, and an agreement does not exist even among researchers. Besides, the word intelligence trend to have a positive connotation, so a broader interpretation must also be neutral because the mentioned ability does not have only good ends (Tegmark, 2017). We are also adopting this definition to include different understandings and to cover all types of intelligence that are non-comparable and quantifiable only by an ability spectrum across goals.

Surpassing the discussion regarding intelligence, another dilemma arises. The lack of consensus does not involve only the definition, and researchers still do not agree if a universal artificial intelligence, the strong AI or the AI mind, will ever be possible. However, even though the technology is still not smart enough, our understanding of its ethical and societal implications is trivial. In the meantime, the current scenario shows that unintended consequences may happen more often than expected, despite good intentions (Coeckelberg, 2020). That is why it is necessary to develop an AI ethics field dedicated to certifying machine behaviors will be ethically acceptable. In this sense, the state-of-the-art ambition of AI ethics would be to create machines able to decide ethically by themselves. And leaving behind the discussion of how machines would gather it (the technical part), knowing what is ethical connects the AI field again with its philosophical branch (Anderson and Anderson, 2011).

Although it is essential to discuss the technology’s future and the possible impacts of strong AI, when we take a realistic view, the focus is inevitably on weak AI since this is the only type we have today. Also, to understand the boundaries of the discussion of AI’s uses and impacts, one might comprehend that this technology can take numerous forms and is a portion of larger technological systems. Some implications and negative outcomes might also regard other technologies (Coeckelberg, 2020). From this standpoint, AI, like any other technology, is one kind of instrument used by humans. Just like the ones from the first machine age, when machines substituted the Human’s muscle power (Brynjolfsson and McAfee, 2016), which is evident when interpreting it as an AI partner. For machines understood as an instrument, humans are responsible for all kinds of outcomes, whether positive or negative. It means all trade-offs and moral dilemmas drawn in rely upon them, so the liability is easy to trace back. It means the ethics differ from the ones toward other intelligent entities since AI is an artifact of our culture and the result of our intelligence (Bryson, 2010).

On the other hand, ethics for humans using machines from the instrumental perspective is not enough for areas that AI is getting into, so we urgently need to discuss ethics for machines. While strong AI is not possible yet, and ethical machines are just an ultimate goal, AI contrasts with traditional technologies by seeking to imitate human behavior. In this regard, the perspective on analyzing ethics has inevitably changed, and we must see it in the content (Anderson and Anderson, 2011). Even though machines are not entirely human-like, what happens when decision-making is solely on the machines? And more, considering all the inconclusive discussions regarding conception and boundaries may turn the technology into a black box where consequences are not completely mapped. We live in the second machine age, and machines are substituting our mental power (Brynjolfsson and McAfee, 2016). Thus, to answer this question, one might deliberate on the changes caused by AI that are reshaping how people interact and flourish while improving our lives (Kim and Mejia, 2019) since AI is getting into domains known as human exclusivity. That said, ethics is one of the features of human life that should be reconsidered to guarantee that machine outcomes will satisfy society’s ethical expectations. As a starting point for this revaluation, we suggest analyzing three critical points to guide the debate - autonomy, explainable AI, and value alignment -, although one may understand that the discussion is not limited to them. However, they emphasize that ethics must be in machines’ content and are clear examples to expose why we must rethink ethics to fit it in the new scenario imposed by AI.

### 2.1. Autonomous, but ethical

If artificial intelligence systems keep increasing their levels of aptitude and penetration in our lives, the worries concerning autonomy will intensify. The preoccupation comes from the fact that autonomous agents are generally the ones deciding freely, without external and ethical constraints (Hooker and Kim, 2019b). This sense of self-law can create a perception of an autonomous AI that could control our future and be our master instead of serving us (Kim et al., 2021) or convert to a “law unto themselves” (Hooker and Kim, 2019b, p. 1). Nevertheless, the point when technology will offer machines capable of intentional agency and skilled enough to settle principles and motivations to guide their own acts and decisions is still in the indefinite future (Hooker and Kim, 2019b). Furthermore, we argue that autonomy is inadequately interpreted as freedom and free will in AI’s circumstance, considering that free will is a central feature of agency mandatory for actions morally responsible (Stanford Encyclopedia of Philosophy, 2018). By understanding what autonomy for machines means, as well as its limitations, ethical questioning will be cleared.

Autonomy’s concept has been studied across fields, including philosophy, psychology, and, more recently, automation technology. The most known concept of autonomy regards free will. Still, behaviorists see it as responses to environmental stimuli, and other descriptions associate it with self-governance and self-control too. Anyway, these earlier focuses differ from the technological ones seen on artificial intelligence, which is related to the autonomous work function without intervention and might sometimes be the connection between humans and machines. In this last overview, autonomy represents the exchange of control from humans to automation (Beer et al., 2014); and the autonomy level varies by the amount of intervention needed (Desai and Yanco, 2005). That said, autonomous agents should be seen as those who hold goals and act on the environment following motivations and a plan not imposed or adopted by other agents, with different levels of intervention. In addition, the idea of no constraints is not successful when it leaves behind the rationality element necessary in the intelligent demands of AI systems. Yet, the rationality needed recalls the long-lasting known principle of ethics for the coherence of the reasons (Hooker and Kim, 2019b).

Within the field of AI, autonomy is often suggested as a feature of human-like or socially interactive. Also, it is related to the capacity to alter its own actions, although just in the environment setting. The term goal is usually connected to this capacity, and control is used inconsistently but is better understood as the lack of intervention (Beer et al., 2014). Nonetheless, one can see that, although it seems AI is deciding by itself, the outcomes are just a reflection of external forces (Etzioni and Etzioni, 2017) given by inputs sent from the environment and following the programming developed by humans. While these characteristics may open space for unethical behavior, it is under discussion that an autonomous action must be explained with coherent reasons, and ethical principles are necessary conditions for it. By setting these frontiers in AI systems, one may see how autonomy does not mean unethical (Hooker and Kim, 2019b) and why machines will not master us like in science fiction. Since people own them, we are the ones determining their goals, as well as their actions and behaviors. Real people are dehumanized by aiming for human-like autonomy, and decision-making is encouraged in poor allocation of resources and responsibility (Bryson, 2010). In other words, the science fiction scenery should not imply apprehension. Despite AI’s autonomy, we set the ethical principles and rationale behind the technology. And, by not having free will, machines should not change their plan settled in the first instance by humans.

While the lack of free will is not a problem if we interpret autonomy for machines in this logic above, one may not omit that the impossibility of being free is also the critics’ argument against artificial consciousness (Casebeer, 2020). In this sense, the autonomy discussion is incomplete if we do not broach the matter to the machine’s conscience. Firstly, one must understand the concept of consciousness, which is, broadly, the awareness of its own existence and can be leveled from a basic and rudimentary sense of self-existence to a reflexive capacity of consciousness. The advanced stages of consciousness still seem too far from being achieved in AI, especially when talking about human metacognition and volition. On the other hand, conscience is the capacity to judge right or wrong, and consciousness is the precondition for it, but it evolves over time, which means some actions accepted before can be considered inappropriate nowadays (Meissner, 2020). In the meantime, we expound that even though advanced levels of consciousness are still not achieved, some kind of conscience is mandatory to fulfill ethical demands on current and expected levels of autonomy in AI systems. From this perspective, the efforts to understand the nature of consciousness in the AI context have created a field known as artificial consciousness (Reggia, 2013). Substantial advancements can be seen in the literature, of which some insight can be seen in Eldeman et al. (1992), Franklin and Graesser (1999), Safron (2022), Cleeremans (2005), Baars and Franklin (2009), Seth (2009), Gamez (2012), Bringsjord et al., 2015, Reggia et al. (2016), Tononi et al. (2016), and Dehaene et al. (2021), just to mention some of them.

Still, the consciousness process is a mystery (Reggia, 2013) and machines’ conscience requires a more profound discussion to show all perspectives and views for and con.^2^ For all that matters in this paper, the autonomy questioning reflects the problem of freedom and determinism (Hooker and Kim, 2019b). While deterministic principles prevent agents from having freedom, some compatibilist principles would reject the idea that freedom is connected to the morally responsible agency since we also have conditioning by our choices and situational variants (Stanford Encyclopedia of Philosophy, 2018). That said, the autonomy notion for AI creates a criterium to distinguish action from behavior and agents from non-agents. Unlike actions, autonomous behavior can give ethical outcomes because it is coherent, respects other agents (Hooker and Kim, 2019b), and is conditioned to the rationality imposed by humans in the first instance. Also, the generalization principle assures autonomy in the interchange that bounds humans and machines since both are seen as agents, although still different ones.

In this reasoning, AI is a system (or embedded in one) that can be considered an autonomous agent since it senses the context to act based on it (inputs) to pursue its own goals, affecting the senses in the future (output) (Franklin and Graesser, 1996). In other words, to develop autonomy while being ethical, machines do not need to acquire people’s feelings since agency and actions governed by an agenda could give the conditions to guide the performance accordingly to each circumstance (Hooker and Kim, 2019b); nor do they need to have consciousness or conscience since moral judgment can be reached from the ethical guidelines given on the counterfactuals, or the external factor inputs provided by humans. Nonetheless, the functioning and interaction between humans and machines must be synergic, and it just happens when trust and reliability are part of the relationship (Dzindolet et al., 2003). So, to satisfy these demands, transparency in explanations becomes essential (Hooker and Kim, 2019b).

### 2.2. Right to explanation and explainable AI

Algorithms autonomously make decisions involving subjects. That said, how the algorithms reach the final decision raises a debate about the explanations as a right to human beings, specifically a moral one. As we exposed in the previous sub-section, autonomous decisions must hold a coherent reason that rational terms can explain. However, aside from the numerous benefits of AI, especially those brought by machine learning, actions and decisions are not always explicable to human users. In addition, machine learning performance has been negatively correlated with explainability. It means that the higher the performance, the less explainable the system is, and the other way around. In this regard, many researchers are working on creating designs whose learning outcomes and decisions are easily comprehended and trusted, as well as to manage the AI’s new generations and keep performance (Gunning, 2019). Yet, different ideas surround the field, and research groups develop distinctive models. In this sense, knowing which model is more suitable is still challenging (Kim, 2018).

On this matter, Explainable AI is the most recent research goal to satisfy these practical, legal, and ethical expectations. This kind of technology has been called XAI, which is correlated with the use, liability issues, right to explanations, and autonomy, among other examples (Kim, 2018). Aiming to provide accountability and transparent systems, the right to explanations is a promissory instrument for governments and other organizations (Wachter et al., 2017a). As a moral right, the right to explanation exists beyond the final result impact, which focuses on protecting users’ privacy in consent transactions and third parties that might be involved in the events (Kim and Routledge, 2018). Nonetheless, explainable AI does not mean just transparent, interpretable, or comprehensive. That is why human psychology has been used to give insights into the required information to create reasonable XAI systems. These requirements regarding what the final users need to understand the decisions to decide on the best application (Gunning, 2019). In other words, besides satisfying ethical expectations, humans still need to comprehend how the decisions were made since machines’ outcomes might be too technical for them.

The debate also concerns explanatory needs, information privacy, and fulfilling legal demands, like the United Kingdom’s GDPR (European Union General Data Protection Regulation 2016/679)^3^ (Kim and Routledge, 2018). However, it is essential to understand how an AI system can offer explanations before discussing human rights regarding this (Wachter et al., 2017a; Kim and Routledge, 2018). Content and timing distinguish the types of explanations. The content relates to system functionality and features, while specific domains include rationale, rules, reasons, circumstances, etc. The time defines if the decision requires an ex-ante (prior) or ex-post (after) explanation. Connecting them, the same way that rationale cannot precede the decision, it is possible to follow that the ex-ante relates exclusively to system functionality (Wachter et al., 2017a; Hooker and Kim, 2019a). From another standpoint, ex-ante is a generic explanation, just like the traditional right to be informed. The ex-post, though, regards specific decisions; they are distinguished by remedial and updating explanations, ensuring that organizations will be fair and responsible when something goes wrong or requests to be reformed (Kim and Routledge, 2018).

Aiming to reach the XAI’s demands, the United States’s DARPA (Defense Advanced Research Projects Agency) program uses three strategies to overcome explainability challenges while maintaining performance. The strategies are deep explanations, which modify deep learning by aiming for explainable features; interpretable model techniques, used for learning more structured and causal models; and model induction, to infer the explanation from any models, such as in the case of black boxes (Gunning, 2019). In this sense, one can easily understand the explanation as the exposition of the decision’s logic. However, literature has argued that, for algorithms, the description related to the external facts that lead to that decision is also necessary. These descriptions are known as counterfactuals (Wachter et al., 2017b) and can be expressed in natural language to provide an intuitive and efficient tool for analyzing machine decisions (Hendricks et al., 2018). In this context, natural language means our language, not algorithms’ mathematical and logical language.

Since many models are being developed, knowing which is good enough is benefitted from the philosophy of science literature that explains the correct versus the excellent explanation. Two major categories of scientific studies are helpful as a starting point: the non-pragmatic theory of the correct answer to a question and the pragmatic view that seeks to give good answers to the audience’s questions. While the non-pragmatic explanation is the most appropriate to the technology demands, human users still need to understand it (Kim, 2018). In other words, an XAI must be explained precisely and deliver good answers without the inaccuracy found in usual pragmatic explanation theories, also not leaving behind ethical expectations. A deeper investigation would also benefit from the theory of knowledge, in which the conditions for knowledge are truth, belief, and justification. Some thinkers also include safety and sensitivity (Wachter et al., 2017b). From this perspective, it is possible to realize that the dialog regarding explainability shows that AI’s problem relies mainly on the lack of one or more conditions of knowledge. Following this idea, a distrust in value misalignment emerges since it is unknown whether machines may be acting unintentionally or carelessly against us and if the outcomes are really going to follow the rationality settled as the condition for being ethical while autonomous.

### 2.3. Value alignment: allying with our values, not theirs

As we suggested in the previous subsection, autonomous machines need the coherence and rationality of reasonable explanations compatible with human values to be ethical. In this regard, the uncertainty around this compatibility is growing (Kim et al., 2018; Kim and Mejia, 2019) as long as highly developed technologies advance in areas considered human exclusivity. This preoccupation reassembles Alan Turing’s ideas of machine adaptation to human standards (Kim et al., 2018). More recently, black-box models and some machine-learning features considered “in the wild” have increased the apprehensions about our security and commitment to society’s values. Nevertheless, many researchers still believe in the potential to develop reliable systems to follow what they are meant to do and what they should not do, aligned with our values (Arnold et al., 2017, p. 1). We already know that machines becoming evil robots is science fiction, though misaligned intelligence is a fact, and the worries about value compatibility rely on it (Tegmark, 2017).

In this scenario, researchers are looking to imitate moral intelligence, not just logic and strategy. These efforts have been grouped as value alignment (VA), and this search seeks to overcome the step to turn machines into moral agents and take AI to a higher level. To reach this goal, machines could learn human preferences or learn ethics (Kim et al., 2019). Inverse reinforcement learning (IRL) is a method that could provide it. The IRL in AI systems would infer preferences from humans (Kim et al., 2018) in order to learn how to work and behave ethically as far as applying rules is too strict to the number of domains and could affect autonomy (Arnold et al., 2017). Anyway, experience shows that machines might learn from biased data engendered by humans (Hooker and Kim, 2019a); consequently, putting human flourishing at the center is no easy task (Kim and Mejia, 2019). Besides, reinforcement learning puts a load too large on the agents that need to evaluate ethics and social character. Also, there are many technical challenges to overcome, and knowing who trains the machine and how the ethics evaluation would happen in action may also be problematic (Arnold et al., 2017). In addition, empirically observing values in human behaviors might mistake an “ought” for an “is.” Simply put, people assume some behaviors as ethical, but it does not necessarily mean they really are (Kim et al., 2019).

Notwithstanding, many challenges emerge in the attempt to make machines learn ethics from humans. This mimetic process is dangerous since people do not always have the best behavior; one may see that they are not always keen to adopt all values observed in their peers. In this regard, an anchored or hybrid model could be more suitable as far as intrinsic values placed into normative concepts could guarantee the alignment (Kim et al., 2018) but without imposing constraints on what is learned empirically (Kim et al., 2019). Norms are safety dispositive to allow decisions without unexpected outcomes resulting from learning through trial and error. That said, ethical and moral values, as well as legal demands, must be principles of the decision-making designed as part of the system to provide more transparent and accountable outcomes, and to reach these outcomes, the intentions, reasons, norms, and counterfactuals must be considered. These conceptual layers show that society evaluates behaviors by seeing if the intentions are antecedent of the actions and are relevant, the reasons underlie the arguments, norms reflect how society expects to correspond, and the counterfactuals place the action into the context (Arnold et al., 2017).

But, even if we develop machines aligned with our values and able to decide with the ethics expected, it is under discussion if machines will ever be moral agents. Moral intelligence, or the ability to determine ethically, is a distinctive element of human intelligence. Moral sensitivity is within the human conscience, which, for instance, is part of the dynamic moral reasoning that repeatedly balances ethics with empirical observation (Kim et al., 2019). In this sense, we might be over-evaluating AI’s potential as it is still impossible to apply all human morality frameworks to machines. Unexpected consequences are evident in putting the technology under low-specified and poorly defined goals or opening space to let its ability to change and create plans result in actions inconsistent with the intention previously projected (Vamplew et al., 2018). That said, joining these three critical points, it is possible to conclude that autonomy differentiates AI from traditional technologies. However, the explainability and alignment demands show that the agency acquired does not give the freedom and free will required to equalize it to the human moral agency. Consequently, we face an artifact that does not fit in any type of agency known, so we must define it to understand better how our ethical frameworks will work in this new configuration.

## 3. Discussion: Moral subjects or moral agents? something in the middle!

The debate about AI’s agency is not new and remount to the 1960s (Taddeo and Floridi, 2018). The instrumental use of machines puts technology in the position of a moral subject, which is characterized as a subject of moral motivation but cannot be held responsible for its own actions (Rowlands, 2012). However, as we exposed in the previous sections, artificial intelligence is changing the old instrumental perspective of ethics surrounding human use to include ethics in the AI’s content. Now, ethics must guide machines’ behavior toward humans and other machines, since they are the agents in these decisions and actions (Anderson and Anderson, 2007). The agency is evident, for example, in the first critical point mentioned before, the autonomy, if we adopt the compatibilism perspective that freedom is not necessary for morally responsible actions. Nonetheless, autonomous machines have been entrusted to many applications, and by dealing with various tasks, the responsibility for outcomes enhances the concerns regarding ethics and security. While dealing with problems just after the occurrences is not enough, explainable AI needs to predict outcomes (Taddeo and Floridi, 2018), not just explain them. In addition, we argue that value alignment should go deeper toward a solution to ensure that the delegation to the autonomous system is also responsible, as well as the fact that VA is proof of why AI’s system may permanently be bonded to humans, does not matter the level of autonomy achieved.

Initiatives are trying to set AI boundaries in society. Academia, government, and the private sector are proceeding toward incorporating ethical principles in modern technology systems, such as reliability, transparency, and accountability (Cooley et al., 2023). One example is the Global Initiative on Ethics of Autonomous and Intelligent Systems (IEEE), which highlights that technology should promote well-being and human flourishing instead of the approach that creates principles and constraints (Vamplew et al., 2018; Kim and Mejia, 2019). The mission of IEEE is not easy since identifying ethical principles to regulate, design, and AI uses bumps into many cultural contexts and domains (Taddeo and Floridi, 2018). Also, to understand the new configuration of ethics and technology, we must consider machine ethics as the field inside AI’s research to analyze ethics dimensions (Anderson and Anderson, 2011), where the central ambition is to make artificial intelligent systems explicit ethical agents, which can calculate the best option in moral dilemmas. However, the challenge is generating this behavior, considering that ethics, even for humans, is still evolving (Anderson and Anderson, 2007).

Humans, as agents, are capable of representing moral norms (moral core), making moral judgments associated with emotions (moral cognition), regulating these emotions and prosocial actions (moral action), and responding to moral criticism by justifying them (moral communication) (Cooley et al., 2023). Artificial Intelligent systems, on the contrary, are autonomous agents different from other technological systems because they can sense the environment and act upon it. The acts follow an agenda, regardless if other agents set the goals firsthand (Franklin and Graesser, 1996). Yet, these autonomous agents expressed in AI systems have been used in a wide range of domains (Awad et al., 2018; Kim, 2018; Hooker and Kim, 2019a; Anagnostou et al., 2022; Ashok et al., 2022; Miller, 2022; Munn, 2022), raising questions about how much we can trust in its moral decision-making and actions, in before exclusively human activities (Cooley et al., 2023). In other words, we reason whether AI technologies have been poorly allocated in moral decision-making domains in which their autonomous agency is insufficient to complete the task.

To understand the background of AI’s agency discussion, we would like to recall James Moor’s three types of agents: the implicit, the explicit, and the full ethical agents. For the implicit moral agents, ethical norms constrain actions because of values embedded in the systems. The explicit one is not so deterministic since it is expected to be an ethical operating system that can respond in moral ways on its own. The full ethical agent is what we recognize as the human moral agency; however, it is not so obvious to understand it. This last type preserves intuitions such as sentience, consciousness, and capacity for suffering, turning them into moral agents and patients. From this point of view, ethical AI systems are moral by not being immoral since they cannot be morally patient and be responsible for acts and consequences (Gamez et al., 2020). In this regard, AI ethics can benefit from the new ethical theories that consider the distributed agency. Traditional ethical frameworks speak to individuals, and human responsibility allocates positive or negative retribution based on individual actions and motivations. But distributed agency implies responsibility shared among all the actors, which is the case of AI and, for example, designers, developers, companies, users, and software/hardware (Taddeo and Floridi, 2018).

The moral agency has been expanded to include partnerships and organizations, for example, but these are still centered in humans when we analyze agenthood. Now, the agency should be stretched to fit the artificial types, which is also essential to understand new moral problems in the machine, general, and mainly distributed ethics (Floridi and Sanders, 2004). At this point, we would like to explain why we consider that AI has been creating a new type of agency in the middle of what we comprehend for subjects and moral agents. Autonomous capacities are living behind the old technology position of moral subjects. However, artificial intelligence is an artifact of our culture (Bryson, 2010), so it is an instrument that must follow our values; that is to say, “machines are ultimately tools of the human beings who design and manufacture them” (Etzioni and Etzioni, 2017). In this sense, it is an implicit ethical agent, following Moor’s typography, that should be constrained by norms in a broader sense to satisfy society’s expectations. Since this deterministic position is not enough for every single decision or outcome, it is necessary to combine features of explicit ethical agents with an ethical operating system. To the explicit ethical part, we argue that the distributed agency could be a key to solving the responsibility problem of the discussion about ethics for machines.

In other words, we suggest a hybrid model that comprises a macro set of norms and rules to guide the system in general terms, reflecting the Moor’s implicit ethical agency but also allowing space to respond in moral ways on their own. Since machines cannot reply to their acts and consequences like humans (Gamez et al., 2020), we explain why the agency should be distributed. This kind of distributed agency relies on theories of contractual and tort liability or strict liability. It separates the intention from the given action or the ability to control outcomes, which is helpful in the case of AI. All agents will hold the responsibility in this distributed system as designers, regulators, and users, also avoiding evil and fostering good by nudging agents toward responsible behaviors (Taddeo and Floridi, 2018). Suppose that technology is putting in society’s hands the power to flourish or destroy itself (Tegmark, 2017). In that case, we should choose the first option by permitting the coexistence between AI and humans to develop better people’s capacities (Hooker and Kim, 2019a,b). Thus, when applying the hybrid agency model for AI, we could think about how to relate it with ethical approaches to find a way to make technology correspond with society’s expectations and people’s flourishing.

Actually, we suggest that the best way to discover how to apply our ethical theories for machines is to clarify the agency. Our argument is that ethical theories aim for individuals and their motivations, moral cognition, and sensitivity; in other words, they aim for individuals with full moral agency. On the other side, AI has brought a scenario where ethical decisions must be made, but different from what is done by humans; that said, after understanding what type of agency applies to AI systems, it will be possible to analyze how ethical theories can fit in. In this sense, we expose that, as an implicit ethical agent, moral principles should be programmed into AI programs. Defenders of this kind of top-down approach (Etzioni and Etzioni, 2017), such as Wallach and Allen (2009), support that ethical choices would be guided by the moral philosophies implanted into the system. Nonetheless, machines do not cope well with vague situations and all nuances humans usually face, whether using one or a combination of moral theories. The other option, the bottom-up approach, accepts that machines could learn ethics by observing humans (Etzioni and Etzioni, 2017). Still, in this kind, there are critics like the naturalistic fallacy, which explains that we can not assume that what is done is what is right (Kim et al., 2019).

By analyzing these challenges, one may see that the troubles are not technological as they reflect old human questionings regarding ethical theories. From this point of view, when appreciating ethics in the AI context, pluralism, which assumes that it is not necessary to choose only one theory of normative ethics (von der Pfordten, 2012), is a valuable alternative to comprehending a hybrid agency model. For instance, deontology and utilitarianisms are formal theories that could help to build an AI Ethics field to develop norms to regulate and guide the implicit ethical agency feature. At the same time, virtue ethics could lead the agents in the distributed agency that would answer to the explicit ethical agency aspect. Virtue ethics, as one of the highest of humans’ purposes of using machines, is a way to avoid moral schizophrenia^4^ of being moved by beliefs that would not seek people’s flourishing. On this matter, we consider that, in the AI field, long-term concepts have been used in new situations, the same way as in other areas, like organizational studies. Still, the concept’s displacement must be appropriate and avoid intellectual traps, such as reductionism (Ramos, 1981), and the same applies to this new situation regarding AI.

In this logic, when machines develop the ability to decide by themself which is the best path to choose will be the turning point to turn them into explicit agents. For example, an autonomous vehicle can navigate by following norms that respect local traffic laws and simple rules like “do not hit and kill people,” which could be expressed as a top-down approach that follows deontology, a normative perspective with criterion-satisfying rationality (De Colle and Werhane, 2008). However, in exceptional situations where there is no other choice than choosing between hit in one person or five people (such as the trolley case^5^), we face a problem where goal-directed rational behavior is best suited (De Colle and Werhane, 2008). That said, the autonomous vehicle could make a utilitarian choice seeking the greater good if this is what local culture most accepts or if it is what has been learned from its owner from the first instance (ethics bots^6^). We, humans, do this all the time; but we take responsibility for our choices, can (most of the time) explain them, and pay for them. Thus, that is our argument for using practical ethical philosophies, like virtue ethics, since they fundament the deliberation part of an ethical decision. In this regard, deepening this discussion in philosophical arguments combined with practical situations is a demand for future research to solve these complex situations.

Furthermore, another problem regarding moral agency relates to rights. In AI’s context, it brings the question: will moral rights be equalized by making machines moral agents just like humans? At this point, we argue that machines are different from humans because they are agents when responsibility is expected, but they are still instruments when we think about rights. And to show this need, explainable AI and value alignment are examples of how machines should serve us, not the other way around. In this sense, it is essential to highlight that this new use of the words *agency* and *agent* just tries to find ways to better understand and allocate the artificial intelligence technologies in our society. However, it does not mean that machines are agents or act like humans; thus, we are not seeking to equal them at the human level. Our preoccupation is not corrupting these words’ conceptions; this paper’s argument follows that the new context imposed other ways to appreciate moral agency and ethics, even though we need to create a new type to comprehend the current reality. This in-between agent is a hybrid model that uses previous knowledge regarding moral agency, respecting distinctions among technology and humans but balancing the needs imposed, such as explainability and learning ethics, to fulfill value alignment.

We already mentioned that a strong type of AI does not exist yet and may never be possible to develop. In this sense, we agree with Etzioni and Etzioni (2017) and their argument that there are strong reasons to believe that AI, no matter how smart and complex, are partners because they carry out very well some task and perform poorly in others, remembering us their role as our instruments that will probably never master us (Bryson, 2010). In other words, if we recall Kant, humanity is what people have expressed in their rational capacities; people are end-in-themselves. However, besides seeking to develop an AI system endowed with rationality, machines are still a phenomenon and a mere means. In this sense, machines will never be full agents, which is our place; however, our argument takes another path by believing that, no matter how difficult it is to implant some attributes into them, we are creating a technology that is changing society’s configuration and the consequence is the emergence of a new type of agency; that said, how to fit our ethical models into their systems need to be discussed. In conclusion, we do not aim to develop a computational formula to solve ethical questions regarding artificial intelligence or to distinguish ontologically humans and machines. Our argument supports that artificial intelligence made machines become moral subjects, but they are still different from the already known ethical principles. So, to make machine ethics possible, we need to rethink our theories considering the new types of agencies created by artificial intelligence.

## 4. Conclusion

This theoretical essay discussed three critical points that expose how ethics is a demand in artificial intelligence content: autonomy, right of explanation, and value alignment. Although the challenges in the AI field are not limited to them, this argument defends machines as instruments in the first machine age, where ethics was used to guide humans using them. However, the second machine age gave us artificial intelligence and its mimetic processes to be human-like. By doing that, ethics must be part of the systems, and machines must be turned from moral subjects to some kind of moral agents. Anyway, this moral status does not put machines on a human level. Still, it proves that we need to appreciate new ways of seeing ethics and find a place for machines, using the inputs of the models we have been using for centuries but adapting to the new reality of the coexistence of artificial intelligence and humans.

For further research, we suggest investigating hybrid or mixed agency models more profoundly and relating them with ethical models; for this purpose, ontological distinctions between human intelligence and artificial intelligence may be helpful. As we briefly mentioned in the previous section, it would be interesting to interpret deontology and utilitarianism for the implicit ethical agency and virtue ethics to guide distributed agency. However, how to solve this complexity would benefit from a more profound philosophical evaluation, combined with empirical research, taking into account that this pluralistic view still does not answer how we, humans, deliberate and choose one ethical perspective over another since ethics is a branch evolving even for humans. In this logic, AI Ethics could benefit from the experience of other fields already using formal ethical models to create norms and recommend best practices, such as Bioethics. Nonetheless, human flourishing must be the ultimate goal since AI systems are our instruments created by us in order to make our life better. That said, virtue ethics can be a solution to reach the human being behind machines.

## Author contributions

All authors listed have made a substantial, direct, and intellectual contribution to the work and approved it for publication.

## Conflict of interest

The authors declare that the research was conducted in the absence of any commercial or financial relationships that could be construed as a potential conflict of interest.

## Publisher’s note

All claims expressed in this article are solely those of the authors and do not necessarily represent those of their affiliated organizations, or those of the publisher, the editors and the reviewers. Any product that may be evaluated in this article, or claim that may be made by its manufacturer, is not guaranteed or endorsed by the publisher.

## Footnotes

^1^The anthropomorphic illusion happens when people transfer the blurred knowledge regarding humankind and related concepts – intelligence, rationality, consciousness, and sentience, for example – to other entities and things, as far as our language tends to hypostatize ideas by confusing concepts with real structures (Polo, 2006). This illusion was analyzed before by Ramos (1981) in the organizational context, and we suggest that the same logic has been applied to AI studies.

^2^Since our objective is not getting into this discussion now, our position on this topic is that consciousness is one of the unique human characteristics; that is why it is a mystery and unable to be put into technical/mechanical parameters. Nonetheless, we believe that, despite the lack of free will and all the unsolved questions regarding our consciousness and conscience, it is necessary to discuss new ways of deliberating over it to make room for the new scenario imposed by AI. The improvements in AI’s autonomy are related to developing some kind of moral judgment and motivation, despite the differences between how humans are conscious of themselves and AI systems interact with the world. In other words, even though we follow a perspective that believes human consciousness is unreachable in technological terms, the AI context requires the conception of new types of consciousness, even if not at the same level or comparable to ours.

^3^It is under discussion whether the European Union General Data Protection Regulation 2016/679 (GDPR) will be able to reach the explainability goal since the protections offered might not be effective enough due to the lack of precise and well-defined discourse. The consequence is that this initiative may just offer the already known right to be informed rather than the aimed right to explanation (Wachter et al., 2017a,b).

^4^Moral schizophrenia was explained by Stocker (1976) as being moved by motive split from reasons, or doing what is bad or being disgusted by what one wants to do.

^5^The trolley case is a philosophical thought experiment that expose a moral dilemma where people need to choose between two undesirable alternatives. It is under discussion if these cases have been misused in context of AI systems (LaCroix, 2022). Anyway, recently, is growing the number of studies highlighting the resemblance between trolley cases and dilemmas faced by autonomous driving (Schäffner, 2020).

^6^Ethics bots are AI programs that capture and learn people’s preferences to instruct machines’ behavior to perform in accordance to them (Etzioni and Etzioni, 2017).
